# Increased risk of cancer in patients with primary sclerosing cholangitis

**DOI:** 10.1007/s12072-021-10214-6

**Published:** 2021-08-06

**Authors:** Aiva Lundberg Båve, Annika Bergquist, Matteo Bottai, Anna Warnqvist, Erik von Seth, Caroline Nordenvall

**Affiliations:** 1 Department of Medicine Huddinge, Karolinska Institutet, Stockholm, Sweden; 2 Division of Hepatology, Department of Upper GI Disease, C1:77 Karolinska University Hospital, Huddinge, 141 86 Stockholm, Sweden; 3 Division of Biostatistics, Institute of Environmental Medicine, Karolinska Institutet, Stockholm, Sweden; 4 Department of Molecular Medicine and Surgery, Karolinska Institutet, Stockholm, Sweden; 5 Department of Pelvic Cancer, GI Oncology and Colorectal Surgery Unit, Karolinska University Hospital, Stockholm, Sweden

**Keywords:** Epidemiology, Matched cohort, National register, Hepatobiliary cancer, Colorectal cancer, Pancreatic cancer, Lymphoma, Inflammatory bowel disease, Ulcerative colitis, Crohn’s disease

## Abstract

**Background and aims:**

Primary sclerosing cholangitis (PSC) is associated with an increased risk of hepatobiliary and colorectal cancer, but the risks of other cancer forms have not been explored. The aim of this study was to evaluate the risk of intestinal and extraintestinal cancers in a large, well-defined cohort of PSC patients.

**Material and method:**

A matched cohort study of Swedish PSC patients was performed with up to ten comparators for each patient, matched for sex, age, and residency. The data were retrieved from national registers. Patients were followed from PSC diagnosis until cancer diagnosis, liver transplantation, first emigration date, death, or December 31, 2016. The risk of cancer was estimated using the Kaplan–Meier method and Cox regression models.

**Results:**

In total, 1432 PSC patients with a verified diagnosis and 14,437 comparators were studied. The mean follow-up time was 15.9 years. Eighty-eight percent of the PSC patients had concomitant inflammatory bowel disease. PSC patients ran significantly increased risks of developing any cancer [HR 3.8, 95% confidence interval (CI) 3.3–4.3], hepatobiliary cancer (HR 120.9, 95% CI 72.0–203.1), colorectal cancer (HR 7.5, 95% CI 5.6–10.0), pancreatic cancer (HR 8.0, 95% CI 3.2–20.2), gastric cancer (HR 4.2, 95% CI 1.5–11.3), small bowel cancer (HR 21.1, 95% CI 3.5–128.2), and lymphoma (HR 3.0, 95% CI 1.6–5.7). PSC was not associated with a lower risk of any cancer form.

**Conclusions:**

PSC patients have a four times overall increased risk of developing cancer compared to the general population, with increased risk of developing hepatobiliary, colorectal, and pancreatic cancer, as well as lymphoma.

**Supplementary Information:**

The online version contains supplementary material available at 10.1007/s12072-021-10214-6.

## Introduction

Primary sclerosing cholangitis (PSC) is a rare immune-mediated bile duct disease closely linked to inflammatory bowel disease (IBD). PSC progresses to end-stage liver disease and the only potential curative treatment is liver transplantation. Life expectancy is limited due to liver failure and increased risk of cancer, which poses a heavy psychological burden on patients [1]. The risk of hepatobiliary cancer in PSC has been shown to be 400–600 times higher than in the general population [2, 3] and can occur at any stage of liver disease. One third of the hepatobiliary cancers are reported as being diagnosed within the first year following PSC diagnosis [2, 4].

PSC is also a risk factor for colorectal cancer or dysplasia in patients with IBD compared to patients with non-PSC IBD [2, 5]. An increased risk of pancreatic cancer has been reported [3, 6–9], but the risk of other cancers in PSC patients has not been thoroughly investigated. Several studies have shown a correlation between cancer and other autoimmune diseases including IBD, where increased risk especially for lymphoma has been described [10–12].

The reason for the high risk of cancer in PSC is unknown but is believed to be associated with the chronic inflammation in the liver and gut. In addition, assessing the risk of cancer in PSC patients is challenging. Many studies originate from tertiary centers, and are limited by their size, retrospective nature, and the risk of selection and referral bias with the possibility of overestimating risks. Population-based studies are justified for the assessment of adequate risk estimates. In rare diseases, such as PSC, cases tend to cluster at specialized centers, which influences the detection rate and true population-based settings are difficult to achieve. PSC patients are rarely followed up in primary care. Hence, large hospital cohorts where PSC diagnosis is confirmed by the scrutiny of medical records is likely to be equally as suitable as a population-based setting for risk estimate assessment.

To further explore the risk of cancer in PSC, we have identified a unique PSC cohort where the diagnosis of PSC was clinically confirmed and the follow-up complete. Considering the autoimmune profile of PSC, we hypothesize that PSC patients have an increased risk of developing several cancers in addition to cancer in the liver and large bowel.

## Methods

### Setting

The register data for PSC patients and general population comparators were obtained from the Swedish National Board of Health and Welfare, and the following registers were used; the Population Register; the Cause of Death Register (1961–2016); the National Patient Register (1969–2016); and the National Swedish Cancer Register (1958–2016) [13–16].

The National Patient Register started in 1964 and has had full coverage since 1987. The register includes data on hospitalizations and contains the following: (i) the patient’s National Register Number (a 10-digit identifier containing date of birth, providing a unique number assigned to all Swedish residents); (ii) hospital admission and discharge dates; and (iii) one main discharge diagnosis and up to 8 additional diagnoses. Information from the National Patient Register includes day surgery from 1997 and outpatient care from 2001. The data from the National Patient Register were used to classify IBD and PSC diagnoses according to the ICD version 8 (1969–1986); ICD-9 (1987–1996); and ICD-10 thereafter (Supplementary Table 1).

The National Swedish Cancer Register was established in 1958 and the recording is nearly complete (98%) [17]. The data on all cancers diagnosed in the cohort between 1958 and 2016 were identified and classified according to ICD-7 to ICD-10. Diagnosis of non-melanoma skin cancer was excluded in the analysis due to its benign course and the quite recent introduction of reporting basal cell carcinoma to the cancer register (2003). The Population Register in Sweden was started in the beginning of the seventeenth century and provides data on residency and migration.

### Study design and study population

We performed a matched cohort study based on data from Swedish National Registers. The cohort consisted of 18,972 individuals: 1768 PSC patients and 17,204 comparators.

PSC patients (*n* = 1768) were identified and recruited from 13 hospitals in nine Swedish cities (tertiary and less specialized centers). Patients’ medical records were scrutinized to confirm the PSC diagnosis (*n* = 1426) [3]. Additional cases with PSC (*n* = 342) were identified through the Swedish Inflammatory Bowel Disease Register (SWIBREG) [18] where PSC diagnosis is registered by a gastroenterologist.

Date and age at the time of PSC diagnosis, presence and type of IBD as well as gender were registered in the database.

Statistics Sweden (SCB) provided up to 10 general population comparators for each PSC patient. Comparators were matched for sex, age, and residency at the time of PSC diagnosis. If date of PSC diagnosis was not registered in the original database, the date for IBD diagnosis was used as the match date (*n* = 337, all from SWIBREG), and in 14 cases, date of birth was used as the match date. None of the comparators were used for more than one PSC patient, and no PSC patient was used as a comparator.

**Fig. 1:**
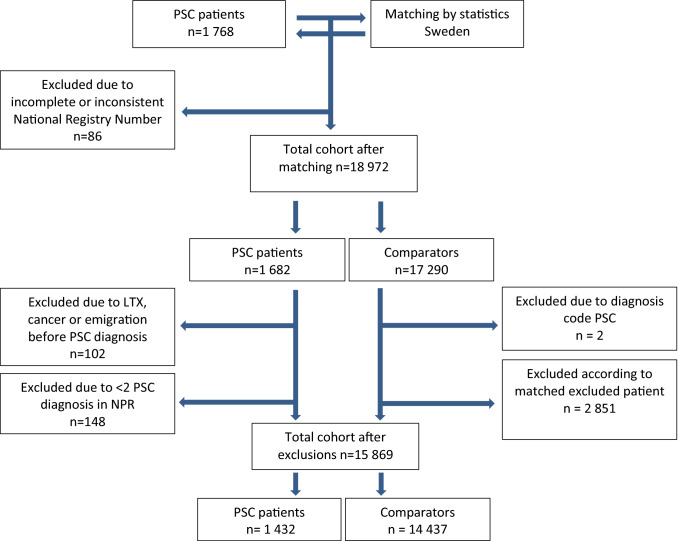
Flow chart describing the cohort

PSC patients with incomplete or inconsistent National Register Number were excluded (*n* = 86) (Fig. 1). To strengthen the accuracy of the PSC diagnosis and to avoid misclassification, we only included patients with a diagnosis code of cholangitis or PSC on two separate occasions in the National Patient Register (Supplementary Table 1). PSC patients without codes for PSC diagnosis on two separate occasions in the register (*n* = 148), or with a record of cancer, transplantation or emigration date prior to the start of the follow-up (*n* = 102) were excluded, as were their corresponding comparators. Two of the comparators had a diagnosis code of PSC in registers, and these individuals were excluded from further analysis.

IBD was defined as having two separate diagnosis codes (Supplementary Table 1) of IBD in the National Patient Registers.

Patients were censored at the time of liver transplantation, and; therefore, no cancers diagnosed after liver transplantation were included. The final cohort consisted of 15,892 individuals, 1432 PSC patients and 14,437 comparators.

### Statistical analysis

Differences in the distribution of the categorical variables between PSC patients and comparators were tested with Pearson’s chi-squared test. The cohort was followed from date of PSC diagnosis (i.e., matching date) until cancer diagnosis. For those where matching date was not the date of PSC diagnosis, follow-up started at the time of first PSC record in the National Patient Register. The outcomes of interest were cancer diagnosis, and censoring events were liver transplantation, emigration, death, or December 31, 2016. The Kaplan–Meier method was used to describe the cumulative incidence of any cancer, and specific cancer types in PSC patients and comparators. In the overall sample and in the subgroup analyses, we estimated the crude incidence rates. The hazard ratios (HR) and their 95% confidence intervals (CI) in PSC patients and comparators were estimated using the Cox proportional hazards model.

To decrease the risk of detection bias, two sensitivity analyses were performed. In the first one, follow-up started one year after PSC diagnosis (i.e., matching date).

In the second sensitivity analysis, the cohort was followed from date of PSC diagnosis/matching date until the first cancer diagnosis.

Data analysis was performed using Stata 15 (Stata Corp, College station, TX). Ethical approval was acquired from the Regional Ethical Board, Stockholm, Sweden (Ref no.: 2016/2343-31/4, and 2017/2041-32).

## Results

### Descriptive data

The final study cohort consisted of 1432 PSC patients and 14,437 comparators. The majority of PSC patients, 69%, were males (Table 1). The median age at PSC diagnosis was 31, and the median year of PSC diagnosis was 2000. Only 53 individuals (4%) were diagnosed with PSC at the age of 60 or above. IBD was found in 88% of the PSC patients, ulcerative colitis (UC) being the most common subtype of IBD (84%). Few of the comparators had received a diagnosis of IBD (1.4%). The total follow-up time for PSC patients and comparators was 253,055 years with a median follow-up per individual of 14.5 years (mean 15.9). Among the PSC patients, 221 were liver transplanted, 17 emigrated and 99 died during follow-up.

**Table 1:** Clinical characteristics of PSC patients and their general population comparators

	PSC patients		Comparators	
	*N*	%	*N*	%
All	1432	100	14,437	100
Female	447	31.2	4388	30.4
Male	985	68.8	10,049	69.6
IBD	1261	88.1	199	1.4
UC	1043		125	
Crohn	207		66	
IBD-U	11		8	
PSC	1432	100.0	0	0.00
Age at PSC diagnosis				
Median (range)	31 (3–80)			
0–19	289	20.2	2935	20.3
20–39	697	48.7	7164	49.6
40–59	393	27.4	3857	26.7
60–80	53	3.7	481	3.3
Year of PSC diagnosis				
Median (range)	2000 (1969–2016)			
1969–1986	197	13.8	1956	13.6
1987–1996	379	26.5	3860	26.7
1997–2005	374	26.1	3894	27.0
2006–2016	482	33.7	4727	32.7
Censoring during follow-up				
Liver transplantation	221	15.4	1	0.01
Emigration	17	1.2	337	2.3
Death	99	6.9	543	3.8
End of follow-up	825	57.6	12,479	86.4

### Outcomes

#### Overall risk of cancer

**Fig. 2:**
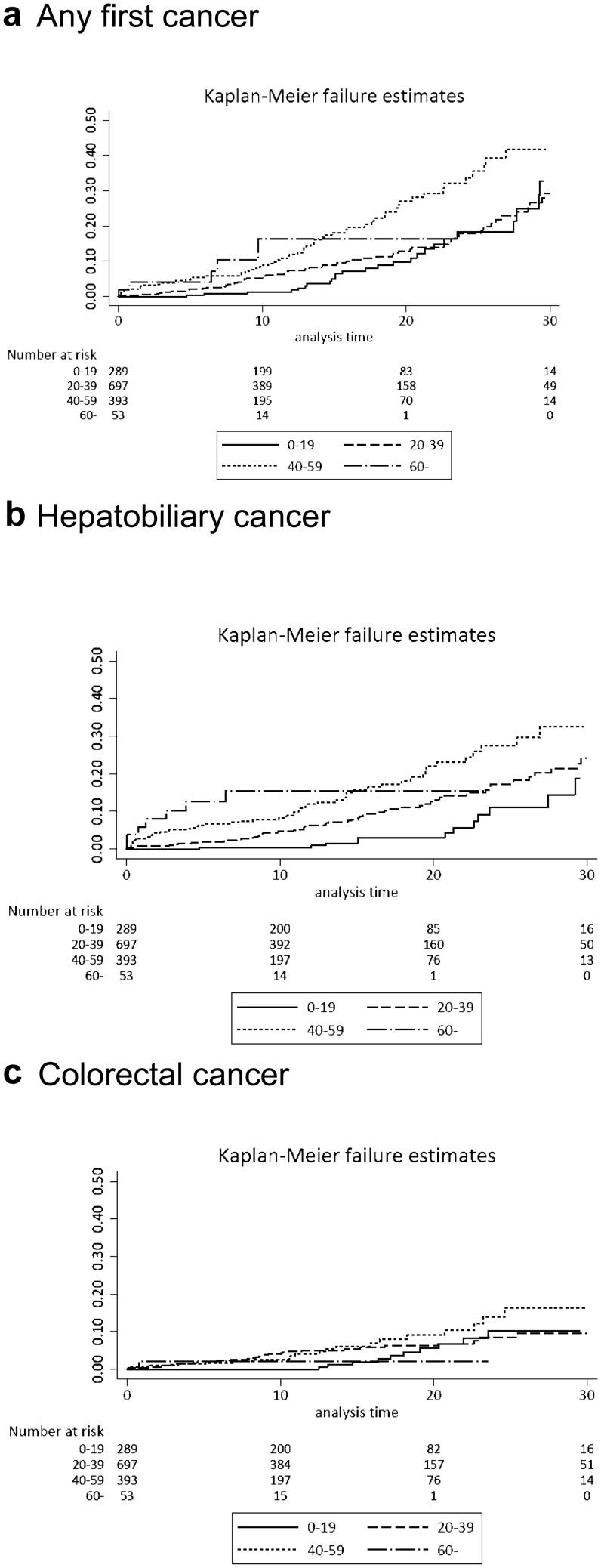
Cumulative cancer risk by age at diagnosis in PSC patients. **a** Any first cancer, **b** Hepatobiliary cancer, and **c** Colorectal cancer

During follow-up, 318 of 1432 PSC patients were diagnosed with cancer, of whom 28 patients were diagnosed with two cancers, and two patients with three different types of cancer. Seven PSC patients were diagnosed with both hepatobiliary cancer and colorectal cancer. The overall risk of developing any first cancer was increased in PSC patients (HR 3.8, 95% CI 3.3–4.3) (Table 2), with a cumulative incidence of 12%, 27%, and 45% at 10, 20, and 30 years after PSC diagnosis. The risk was more pronounced in patients over 40 years of age at the time of PSC diagnosis, but no difference in risk was seen in men versus women (Fig. 2, Supplementary Table 2).

**Table 2:** Risk estimates demonstrated as hazard ratios (HR) with 95% confidence intervals (CI) and absolute rate difference (ARD) of all cancers and stratified by cancer type in PSC patients (*n* = 1432) and general population comparators (*n* = 14,437)

Cancer type	PSC patients		Comparators		ARD	HR	95% CI
	*N*	%	*N*	%	*N*/100,000		
Any first cancer	285	19.9	1085	7.5	1111.1	3.8	3.3–4.3
Hepatobiliary	140	9.8	16	0.1	732.7	120.9	72.0–203.1
Colorectal	68	4.8	130	0.9	307.9	7.5	5.6–10.1
Esophageal	1	0.07	10	0.07	1.0	1.4	0.2–11.1
Gastric	5	0.4	18	0.1	18.6	4.1	1.5–11.3
Small bowel	3	0.2	2	0.01	14.8	21.1	3.5–128.2
Pancreatic	7	0.5	13	0.1	31.1	8.0	3.2–20.2
Lung cancer	7	0.5	77	0.5	4.4	1.3	0.6–2.8
Breast cancer	8	0.6	116	0.8	− 6.7	0.9	0.5–1.9
Female genital	19	4.3^a^	149	3.4^a^	37.2	1.6	1.0–2.6
Male genital	16	1.6^b^	248	2.5^b^	− 20.2	0.9	0.6–1.6
Kidney and bladder	3	0.2	64	0.4	− 11.1	0.7	0.2–2.2
Melanoma	8	0.6	91	0.6	3.9	1.2	0.6–2.4
Lymphoma	11	0.8	51	0.4	36.3	3.0	1.6–5.7
Myeloma and leukemia	3	0.2	46	0.3	− 3.6	0.9	0.3–2.9
Other	18	1.3	155	1.1	29.32	1.6	1.0–2.6

^a^Percentage of number of females

^b^Percentage of number of males

### Risk of hepatobiliary cancer

**Fig. 3:**
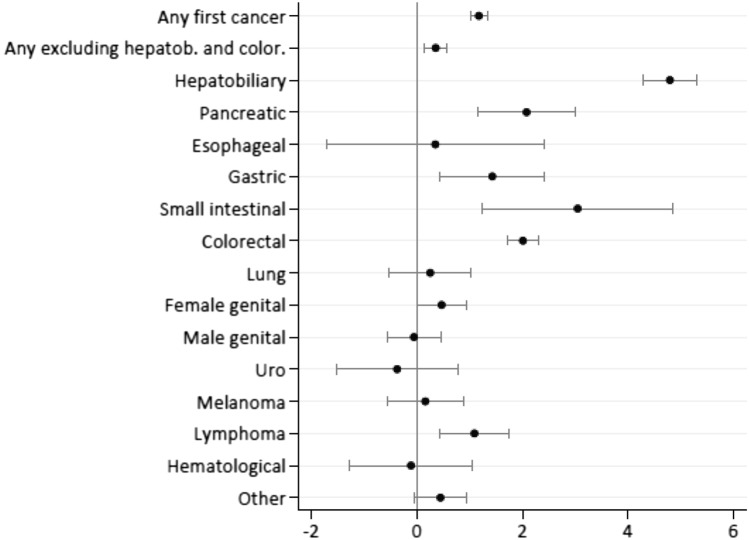
Log hazard ratios of cancer risks in PSC patients when compared with general comparators

During follow-up, 10% (*n* = 140) of the PSC patients and 0.1% (*n* = 16) of the comparators were diagnosed with hepatobiliary cancer, of which, 9% of the PSC patients and 0.1% of comparators received a primary cancer diagnosis of hepatobiliary cancer. The HR for hepatobiliary cancer in PSC patients was 120.9 (95% CI 72.0–203.1) with an absolute rate difference of 733 per 100,000 patient years (Table 2, Fig. 3). The cumulative incidence of hepatobiliary cancer in the PSC patients at 10, 20, and 30 years after diagnosis were 5%, 13%, and 25%, respectively (Fig. 2, Supplementary Table 2). The risk was more pronounced in patients over 40 years of age at the time of PSC diagnosis (Fig. 2, Supplementary Table 2). Only after 30 years of PSC diagnosis, there was a risk difference according to gender with a doubled risk for men of developing hepatobiliary cancer (Supplementary Table 2).

### Risk of gastrointestinal tract cancer

Colorectal cancer was diagnosed in 5% (*n* = 68) of the PSC patients and 1% (*n* = 130) of the comparators, and as the primary cancer diagnosis in 4% of the PSC patients and 1% of the comparators. PSC patients had an increased risk of colorectal cancer [HR 7.5 (95% CI 5.6–10.1)] with an absolute rate difference of 308 cases per 100,000 patient years (Table 2, Fig. 3). The cumulative incidence of colorectal cancer in the PSC patients at 10, 20, and 30 years after diagnosis were 3%, 7%, and 11%, respectively with a higher risk over time for men than women up to 20 years after PSC diagnosis (Fig. 2, Supplementary Table 2).

PSC patients also had an increased risk of pancreatic cancer [HR 8.0 (95% CI 3.2–20.2)], gastric cancer [HR 4.1 (95% CI 1.5–11.3)] and small bowel cancer [HR 21.1 (95% CI 3.5–128.2)], even though the latter two were based on a limited number of cases (Table 2, Fig. 3).

### Risk of extra gastrointestinal cancer

The risk of lymphoma was increased in patients with PSC [HR 3.0 (95% CI 1.6–5.7)], with an absolute rate difference of 36 cases per 100,000 patient years. The risks of other hematological malignancies were not increased.

There was no form of cancer for which PSC patients were at a lower risk than the comparators.

### Sensitivity analysis

The first sensitivity analysis, where follow-up began one year after PSC diagnosis, revealed a similar risk estimate for hepatobiliary cancer as the main analysis [HR 111.9 (95% CI 65.35–191.7)], (Supplementary Table 3).

In the second sensitivity analysis, where only the first cancer diagnosis was considered, no difference from the main analysis was detected (Supplementary Table 4).

## Discussion

The risk of cancer following PSC diagnosis was studied in the Swedish cohort of 1432 well defined PSC patients and 14,437 comparators. During more than 15 years of follow-up, the well-known increased risks for hepatobiliary and colorectal cancer were confirmed. Increased risk of pancreatic cancer and lymphoma were also found. PSC was not associated with a decreased risk of any type of cancer.

Nearly 90% of the PSC patients in our study were also diagnosed with IBD which is a higher than usually described [19]. PSC patients without IBD had a greater cumulative risk of developing any cancer and hepatobiliary cancer, but these numbers need to be interpreted cautiously considering the skewed proportions of IBD-PSC versus non-IBD-PSC.

The risk of hepatobiliary cancer in PSC patients was in line with previous population-based studies, with a 120-fold increased risk, and cumulative incidences of 5%, 13%, and 25% at 10, 20, and 30 years after diagnosis, respectively. In the present study, only 14% of the hepatobiliary cancers were diagnosed during the first year of follow-up which is lower than previously reported [2, 4, 7, 8]. This low figure may be an effect of an earlier diagnosis in more recent years. It is reported that PSC is increasingly detected at earlier disease stages. In addition, the large proportion of IBD patients also contributes to earlier diagnosis since they are likely to be screened with liver function tests on a regular basis. Another explanation may be that patients with cancer prior to PSC diagnosis were excluded in the present study.

Increased risk of colorectal cancer in PSC patients was shown with a sevenfold [HR 7.5 (95% CI 5.6–10.1)] increase in risk. The cumulative incidence of colorectal cancer was lower than previously reported, but similar to results from more recent studies from Denmark and the Netherlands [2, 20].

In the present study, PSC patients showed an eightfold increased risk of pancreatic cancer (HR 8.0, 95% CI 3.2–20.2), which is in line with results from smaller cohorts of PSC patients [3, 6], and studies reliant on ICD codes for PSC diagnosis [7, 9]. The ICD classification for PSC prior to version 10 is unspecific, which may lead to selection bias and nongeneralizable results. We confirmed the PSC diagnosis by scrutinizing medical records, in addition to verifying the diagnosis in the National Patient Register, which is why the risk estimate for pancreatic cancer in our study is based on a robust definition of PSC. However, there is a risk of misclassification since distal cholangiocarcinoma may have been registered as pancreatic cancer. In clinical practice, evaluation of the pancreas should be made in patients undergoing imaging surveillance for early detection of cholangiocarcinoma.

The increased risks of gastric cancer and small bowel cancer, even though numerically significant, should be interpreted with caution. A low number of cases of these cancer forms was detected and may well have been a random finding.

A threefold increase in the risk of lymphoma was detected in this cohort. An increased risk of lymphoma in the post-transplanted setting is well known [21, 22], but has not previously been shown in nontransplanted PSC patients [3, 6], which may be due to lack of prevalence. In IBD, studies on the risk of hematological malignancies have shown contradictive results [23, 24], although increased risk of lymphoma in patients with Crohn’s disease has been discussed [25]. All PSC patients with lymphoma had a concomitant IBD (82% UC and 18% Crohn’s) in this study.

One of the strengths of this study is the high number of well-defined PSC patients from a mixed PSC population and not restricted to tertiary or highly specialized centers, which increases the generalizability. Complete follow-up was achieved through Swedish registers, and no patient was lost in the follow-up. The cancer register has high coverage (98%) [17].

The study has limitations. It is limited by its inability to adjust for cancer risk factors, such as smoking, alcohol, medication, obesity, viral status of Epstein–Barr virus, and family history. There was no information on stage of liver disease. Unfortunately, information on cancer cases was limited to register data, which made it impossible to distinguish between cholangiocellular and hepatocellular carcinoma. The choice of comparators can also be questioned. Comparators were matched for age, sex, and residency but not for IBD. The unique phenotype of IBD in PSC with its specific features may not be comparable to non-PSC IBD [26], which motivates comparators from the general population. On the other hand, risk increase related to IBD could not be evaluated.

In conclusion, PSC patients had a four times increased risk of cancer compared to the general population. The highest risks were evident in hepatobiliary and colorectal cancer, but also in other gastrointestinal cancers and lymphoma. PSC was not associated with a lower risk of any cancer form.

## Supplementary Information

Below is the link to the electronic supplementary material.

Supplementary file1 (DOCX 32 kb)

## Data Availability

Data available on request due to privacy/ethical restrictions.

## Abbreviations

ATC: Anatomical therapeutic chemical
CI: Confidence interval
HR: Hazard ratio
IBD: Inflammatory bowel disease
ICD: International classification of diseases
PSC: Primary sclerosing cholangitis
SCB: Statistics Sweden
SWIBREG: Swedish Inflammatory Bowel Disease Register
UC: Ulcerative colitis

## Code availability

Data available on request due to privacy/ethical restrictions.
