# Supplementary material for: Increased risk of cancer in patients with primary sclerosing cholangitis
Source: Hepatol Int. 2021 Aug 6;15(5):1174–82. doi: 10.1007/s12072-021-10214-6 (PMC8514354; doi:10.1007/s12072-021-10214-6)
Supplement: Supplementary file 1 — Supplementary file1 (DOCX 32 kb) [file 12072_2021_10214_MOESM1_ESM.docx]

**Supplementary table 1**

ICD-codes used for PSC, comorbidities, and outcomes

| **Diagnosis** | ICD 7 | ICD 8 | ICD 9 | ICD 10 |
| --- | --- | --- | --- | --- |
| **Inflammatory Bowel Disease (IBD)** |  |  |  |  |
| Ulcerative Colitis (UC) | - | 563.1 563.9 569.0 | 556 | K51 |
| Crohns Disease (CD) | - | 563.0 | 555 | K50 |
| IBD-Unspecified (IBD-U) | - | - | - | K523 K529 |
| Primary sclerosing cholangitis / cholangitis | 585,29 | 575.05 | 576 | K830A K83.0 |
| Liver transplantation | - | - | 5059 | JJC00-40 DJ005, DJ006 |
| **Cancer** **(all)** | 140-209 | - | - | - |
| Hepatobiliary cancer | 155 | - | 155-156 | C22-C24 |
| Buccal cavity | 140-148 |  |  |  |
| Esophageal | 150 |  |  |  |
| Gastric | 151 |  |  |  |
| Small intestinal | 152 |  |  |  |
| Colorectal | 153-154 |  |  |  |
| Pancreas | 157 | - | 157 | C25 |
| Other intestinal | 159 |  |  |  |
| Lung | 162-163 |  |  |  |
| Breast | 170 |  |  |  |
| Female genital | 171, 172, 175, 176 |  |  |  |
| Male genital | 177-179 |  |  |  |
| Urological (kidney and bladder) | 180-181 |  |  |  |
| Melanoma | 190 |  |  |  |
| Brain | 193 |  |  |  |
| Thyroid | 194 |  |  |  |
| Lymphoma | 200-202 |  |  |  |
| Hematological (Myeloma, leukemia) | 203-207 |  |  |  |

**Supplementary table 2**

Cumulative incidence of any first cancer, hepatobiliary- and colorectal cancer in PSC-patients by age and year at PSC diagnosis, and by sex and concomitant IBD

|  | **Any first cancer (%)** | | | | | **Hepatobiliary cancer (%)** | | | | | **Colorectal cancer (%)** | | | | | |
| --- | --- | --- | --- | --- | --- | --- | --- | --- | --- | --- | --- | --- | --- | --- | --- | --- |
| Years after PSC | 1 | 5 | 10 | 20 | 30 | 1 | 5 | 10 | 20 | 30 | 1 | 5 | 10 | 20 | 30 |  |
| **All patients** | 2.1 | 5.8 | 11.9 | 26.8 | 45.1 | 1.3 | 3.1 | 5.1 | 13.0 | 25.4 | 0.3 | 1.4 | 2.7 | 6.7 | 11.3 |  |
| **Age at PSC** |  |  |  |  |  |  |  |  |  |  |  |  |  |  |  |  |
| 0-19 | 0.0 | 0.4 | 1.2 | 10.6 | 34.9 | 0.0 | 0.4 | 0.4 | 3.0 | 18.7 | 0.0 | 0.0 | 0.0 | 5.6 | 10.2 |  |
| 20-39 | 1.2 | 4.5 | 11.5 | 23.8 | 42.3 | 0.7 | 1.9 | 4.6 | 12.5 | 24.2 | 0.3 | 2.0 | 4.0 | 6.2 | 9.5 |  |
| 40-59 | 4.6 | 10.9 | 19.5 | 44.6 | 57.9 | 2.8 | 6.4 | 8.3 | 22.0 | 32.4 | 0.5 | 1.4 | 2.6 | 9.0 | 16.3 |  |
| 60-80 | 8.0 | 17.1 | 28.7 | 38.9 | - | 5.9 | 12.6 | 15.5 | 15.5 | - | 2.1 | 2.1 | 2.1 | 2.1 | - |  |
| **Year of PSC** |  |  |  |  |  |  |  |  |  |  |  |  |  |  |  |  |
| 1969-1986 | 1.0 | 5.2 | 9.7 | 20.7 | 39.2 | 0.5 | 2.6 | 4.3 | 11.8 | 24.3 | 0.5 | 2.6 | 3.8 | 5.7 | 9.1 |  |
| 1987-1996 | 2.9 | 7.8 | 13.6 | 29.3 | - | 2.1 | 5.1 | 6.6 | 14.1 | - | 0.3 | 1.1 | 2.7 | 7.8 | - |  |
| 1997-2005 | 1.3 | 2.5 | 9.2 | - | - | 0.8 | 1.4 | 3.4 | - | - | 0.3 | 0.8 | 1.4 | - | - |  |
| 2006-2016 | 2.5 | 7.5 | 17.0 | - | - | 1.5 | 3.2 | 6.2 | - | - | 0.4 | 1.8 | 4.2 | - | - |  |
| **Sex** |  |  |  |  |  |  |  |  |  |  |  |  |  |  |  |  |
| Male | 2.2 | 5.3 | 11.7 | 26.7 | 46.1 | 1.3 | 3.0 | 5.4 | 14.0 | 30.2 | 0.5 | 1.6 | 3.2 | 8.2 | 10.7 |  |
| Female | 2.0 | 6.9 | 12.3 | 27.1 | 43.4 | 1.4 | 3.5 | 4.4 | 10.9 | 16.1 | 0.0 | 1.0 | 1.7 | 3.4 | 12.0 |  |
| **Concomitant IBD** |  |  |  |  |  |  |  |  |  |  |  |  |  |  |  |  |
| IBD | 1.9 | 5.2 | 11.6 | 26.7 | 45.0 | 1.2 | 2.7 | 4.7 | 12.9 | 26.0 | 0.4 | 1.6 | 3.0 | 7.5 | 11.4 |  |
| No IBD | 3.5 | 10.4 | 13.7 | 27.3 | 43.7 | 2.4 | 6.7 | 7.5 | 12.9 | 17.1 | 0.0 | 0.0 | 0.0 | 0.0 | 11.9 |  |

**Supplementary table 3**

Cancer risk in PSC-patients (n=1 372) and comparators (n=13 787) with follow-up starting one year after diagnosis

|  | **PSC-patients** | | **Comparators** | |  |  |
| --- | --- | --- | --- | --- | --- | --- |
| **Cancer type** | N | % | N | % | **HR** | 95 % CI |
| All first | 255 | 18.6 | 1 012 | 7.3 | 3.6 | 3.1-4.1 |
|  |  |  |  |  |  |  |
| Hepatobiliary | 120 | 8.8 | 15 | 0.1 | 111.9 | 65.3-191.7 |
| Colorectal | 63 | 4.6 | 121 | 0.9 | 7.5 | 5.5-10.2 |
|  |  |  |  |  |  |  |
| Esophageal | 1 | 0.07 | 10 | 0.07 | 1.4 | 0.2-10.9 |
| Gastric | 5 | 0.4 | 15 | 0.1 | 5.1 | 1.8-14.0 |
| Small intestine | 2 | 0.2 | 2 | 0.01 | 14.8 | 2.1-106.8 |
| Pancreatic | 6 | 0.4 | 12 | 0.1 | 7.6 | 2.8-20.4 |
|  |  |  |  |  |  |  |
| Lung cancer | 6 | 0.4 | 70 | 0.5 | 1.2 | 0.5-2.8 |
| Breast cancer | 7 | 0.5 | 105 | 0.8 | 0.9 | 0.4-2.0 |
| Female genital | 18 | 4.2* | 139 | 3.3* | 1.6 | 1.0-2.6 |
| Male genital | 16 | 1.7** | 234 | 2.4** | 1.0 | 0.6-1.7 |
| Urological | 3 | 0.2 | 60 | 0.4 | 0.7 | 0.2-2.3 |
| Melanoma | 7 | 0.5 | 83 | 0.6 | 1.1 | 0.5-2.4 |
| Lymphoma | 11 | 0.8 | 48 | 0.4 | 3.1 | 1.6-6.0 |
| Myeloma and leukemia | 3 | 0.2 | 45 | 0.3 | 0.9 | 0.3-2.9 |
| Other | 16 | 1.2 | 149 | 1.1 | 1.4 | 0.9-2.4 |

*percentage of number of females **percentage of number of males

**Supplementary table 4**

Cancer risk in PSC-patients (n= 1 432) and comparators (n=14 437) censoring at first cancer diagnosis

|  | **PSC-patients** | | **Comparators** | |  |  |
| --- | --- | --- | --- | --- | --- | --- |
| **Cancer type** | N | % | N | % | **HR** | 95 % CI |
| All | 285 | 19.9 | 1 085 | 7.5 | 3.8 | 3.3-4.3 |
|  |  |  |  |  |  |  |
| Hepatobiliary | 128 | 8.9 | 14 | 0.1 | 124.8 | 71.8-217.0 |
| Colorectal | 62 | 4.3 | 115 | 0.8 | 7.6 | 5.6-10.4 |
|  |  |  |  |  |  |  |
| Esophageal | 1 | 0.1 | 8 | 0.1 | 1.7 | 0.2-14.0 |
| Gastric | 5 | 0.4 | 14 | 0.1 | 5.2 | 1.9-14.5 |
| Small intestine | 3 | 0.2 | 2 | 0.0 | 21.1 | 3.5-128.1 |
| Pancreatic | 7 | 0.5 | 12 | 0.1 | 8.5 | 3.3-21.8 |
|  |  |  |  |  |  |  |
| Lung cancer | 6 | 0.4 | 69 | 0.5 | 1.2 | 0.5-2.8 |
| Breast cancer | 8 | 0.6 | 111 | 0.8 | 1.0 | 0.5-2.0 |
| Female genital | 17 | 3.8* | 146 | 3.3* | 1.5 | 0.9-2.4 |
| Male genital | 10 | 1.0** | 227 | 2.3** | 0.6 | 0.3-1.2 |
| Kidney and bladder | 3 | 0.2 | 52 | 0.4 | 0.8 | 0.3-2.7 |
| Melanoma | 7 | 0.5 | 87 | 0.6 | 1.1 | 0.5-2.3 |
| Lymphoma | 10 | 0.7 | 45 | 0.3 | 3.0 | 1.5-6.0 |
| Myeloma and leukemia | 3 | 0.2 | 39 | 0.3 | 1.0 | 0.3-3.4 |
| Other | 15 | 1.1 | 144 | 1.0 | 1.4 | 0.8-2.4 |

*percentage of number of females **percentage of number of males
